# A web application and service for imputing and visualizing missense variant effect maps

**DOI:** 10.1093/bioinformatics/btz012

**Published:** 2019-01-14

**Authors:** Yingzhou Wu, Jochen Weile, Atina G Cote, Song Sun, Jennifer Knapp, Marta Verby, Frederick P Roth

**Affiliations:** 1 Donnelly Centre, University of Toronto, Toronto, ON, Canada; 2 Department of Molecular Genetics, University of Toronto, Toronto, ON, Canada; 3 Department of Computer Science, University of Toronto, Toronto, ON, Canada; 4 Lunenfeld-Tanenbaum Research Institute, Sinai Health System, Toronto, ON, Canada; 5 Center for Cancer Systems Biology, Dana Farber Cancer Institute, Boston, MA, USA; 6 Canadian Institute for Advanced Research, Toronto, ON, Canada

## Abstract

**Summary:**

The promise of personalized genomic medicine depends on our ability to assess the functional impact of rare sequence variation. Multiplexed assays can experimentally measure the functional impact of missense variants on a massive scale. However, even after such assays, many missense variants remain poorly measured. Here we describe a software pipeline and application to impute missing information in experimentally determined variant effect maps.

**Availability and implementation:**

http://impute.varianteffect.org source code: https://github.com/joewuca/imputation.

**Supplementary information:**

Supplementary data are available at *Bioinformatics* online.

## 1 Introduction

**Fig. 1.**
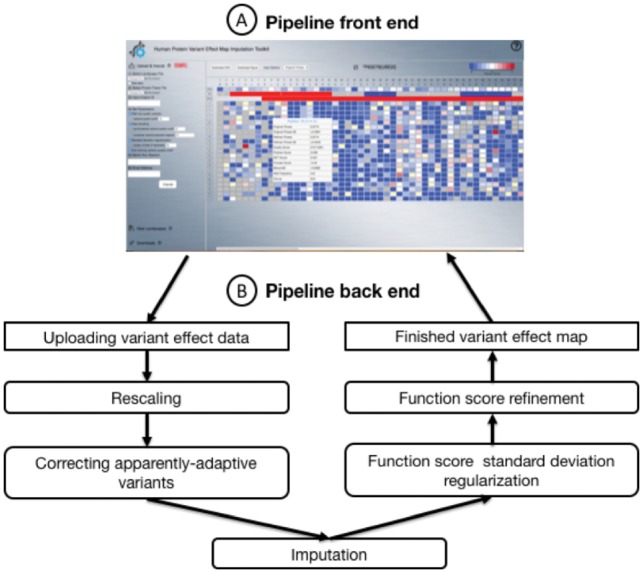
(**A**) The front-end web application. (**B**) The back-end application for data processing and machine learning workflow

Interpreting genome sequences for personalized diagnostics and therapy is becoming increasingly common (Starita *et al.*, 2017). However, our limited ability to interpret which genetic variants are functional has hindered progress. Indeed, among variants in ClinVar that have been subjected to clinical interpretation, the majority has been deemed a ‘variant of uncertain significance’ (Cooper, 2015). Many purely computational methods exist for identifying functional variants, e.g. PolyPhen-2 (Adzhubei *et al.*, 2013), SIFT (Ng and Henikoff, 2001) and PROVEAN (Choi *et al.*, 2012); however, computational methods can detect far fewer disease-associated variants with high confidence than experimental functional assays (Sun *et al.*, 2016). Experimental assays have typically been ‘reactive’, i.e. carried out only after a variant has been observed in a patient. More recently, it has become possible to measure the functional impact of many variants in a single protein using multiplexed assays of variant effect (MAVEs), in which next-generation sequencing is used to measure the effects of functional selection on a mutagenized pool of clones via changes in allele frequency during the selection (Fowler and Fields, 2014, Starita *et al.*, 2015, 2017, Weile *et al.*, 2017, Weile and Roth, 2018). However, some missense variants in MAVE experiments are poorly represented in the mutagenized library so that functional impact cannot be confidently assessed (Supplementary Fig. S1). Previously, we described methods to fill in the missing information in the resulting variant effect (VE) maps, and to refine entries that were poorly measured (Weile *et al.*, 2017). Strong agreement has been found between imputed function scores and individual experimental assays (Weile *et al.*, 2017). Others have used MAVE data to train models for predicting functional impact (Gray *et al.*, 2018), but these models were not optimized for the imputation problem. Here we modify previous computational methods and make them more accessible via a web application. Specifically, we provide: (i) a front-end web application that allows users to upload their own MAVE data and visualize or download a complete VE map (Fig. 1A); and (ii) a back-end data processing service that performs imputation and refinement (Fig. 1B). We note that human protein VE maps (imputed or otherwise) are research tools and should be appropriately validated before clinical use.

## 2 The front-end web interface

The imputation pipeline front end was developed using the web application development tool Google Web Toolkit. The user interface is composed of three sections: (i) Upload and Impute, in which users upload their MAVE data in the appropriate format (See User Guide Sections 5.2 and 5.3), provide the ID from Uniprot (Bateman *et al.*, 2017) that corresponds to their target protein, and select analysis parameters (e.g. quality score threshold). After the back-end application has completed uploading, processing and imputing missing MAVE data, the front end visualizes the complete VE map. (ii) View Landscapes, allowing users to access previously imputed VE maps and revisit user-imputed maps associated with a known session ID. Users may view the landscape of experimentally measured function scores, imputed and refined function scores, or the landscape of scores from computational methods like Polyphen-2 and PROVEAN. (iii) Downloads, where the user can download input data format templates and user guide.

Where no VE map is yet available, the application also allows entry of a UniprotID to retrieve contextual information (e.g. secondary structure annotations) and computational VE predictions from PolyPhen-2, SIFT, PROVEAN. The application is currently limited to the ∼3000 disease-implicated proteins with at least one pathogenic missense variant in ClinVar (Landrum *et al.*, 2018).

## 3 The back-end application

The back-end application was developed using Python and associated ‘scikit-learn’ machine learning package. Upon receiving user-uploaded MAVE data, the back-end application executes a series of jobs (Fig. 1B) to return the complete imputed and refined VE map to the front-end interface so that users can visualize and download the results.

### 3.1 Rescaling function scores

Unless the user indicates that the function scores they uploaded were pre-normalized, the pipeline rescales the score of each variant such that the median of stop codon variants ($Stop_{median}$) is defined to have function score 0 and the median of synonymous variants ($Syn_{median}$) is defined to have function score 1.

$$Rescaled\;FunctionScore=\frac{FunctionScore-Stop_{median}}{Syn_{median}-Stop_{median}}$$

### 3.2 Correcting apparently-adaptive variants

Some variants may appear beneficial, i.e. have greater-than-wild-type function. However, variants that exhibit higher-than-wild-type function in yeast complementation assays are likely to be deleterious in humans (Weile *et al.*, 2017). Therefore, as in Weile *et al.*, function scores X that exceed the wild-type score of 1 are transformed to 1/X.

### 3.3 Modeling MAVE

The application generates a predictive statistical model for the MAVE data. Input features for this model included pre-computed PolyPhen-2 and PROVEAN scores, chemical and physical properties of the wild-type and substituted amino acids, protein structure-related information and the average function score at each position (Supplementary Table S1). (As the automated retrieval of these features may be more generally useful, this is enabled even where MAVE data is not available.) The models are trained using the Gradient Boosted Tree (GBT) method which outperformed other methods (e.g. random forest, SVM and linear regression) for all available VE maps (Supplementary Fig. S2) in 10-fold cross-validation, as measured by root-mean-squared error.

Like the previously-described random forest approach (Weile *et al.*, 2017), a GBT model must be retrained for every new protein entry. Relative to our previously described random forest method, the GBT implementation used in our web application is faster and can also handle missing features in the data. Feature importance for each tree was the number of times a feature was used for splitting, weighted by squared improvement to root-mean-squared error owing to that feature. Average feature importance over all trees was reported (Supplementary Fig. S3). For previous imputation models, the most important feature has been the average function score at each position, with PolyPhen-2, SIFT, PROVEAN and BLOSUM (Henikoff and Henikoff, 1992) scores also being helpful. To include only high-quality measurements in model training, users can either provide a quality cutoff parameter, or let the analysis pipeline select the cutoff that optimizes performance in terms of predicting the test dataset which consists of the top 20% of variants ranked by quality score (Supplementary Fig. S4). The trained GBT model is then applied to each unmeasured missense variant to impute the function score.

### 3.4 Estimating error in function scores

To interpret the function score estimated for a given variant, it is important to understand the uncertainty in that estimate. Given a sufficient number (K) of replicates, the standard error σ for each variant can be accurately calculated from the set of replicates. When fewer than K replicates are available, a regularized estimate of σ is calculated as in Weile *et al*. (2017), updating the measured σ with a prior estimate of σ that is based on an overall regression of σ values against function scores.

### 3.5 Refining measured function scores

The model for imputing missing scores can help refine experimental scores that were imperfectly measured. Refined scores were calculated as a weighted average of imputed and measured scores (weighting by the inverse-square of estimated standard error in each input score).

## Supplementary Material

btz012_Supplementary_Data
