# Supplementary material for: A web application and service for imputing and visualizing missense variant effect maps
Source: Bioinformatics. 2019 Jan 14;35(17):3191–3. doi: 10.1093/bioinformatics/btz012 (PMC6735881; doi:10.1093/bioinformatics/btz012)
Supplement: btz012_Supplementary_Data [file btz012_supplementary_data.zip › btz012-suppl_data/imputation_help_2018_12_04.docx]

**Human Protein Variant Effect Map Imputation Toolkit User Guide**

[1. Upload and impute 3](#_Toc527535293)

[1.1 Select Landscape File 3](#_Toc527535294)

[1.1.1 Raw Data (Checkbox) 3](#_Toc527535295)

[1.1.2 Example – UBE2I and BRCA1 3](#_Toc527535296)

[1.2 Select Protein Fasta File 4](#_Toc527535297)

[1.3 Input Uniprot ID 4](#_Toc527535298)

[1.4 Set Parameters 4](#_Toc527535299)

[1.4.1 Filter low quality missense variants 4](#_Toc527535300)

[1.4.2 Data rescaling 5](#_Toc527535301)

[1.4.3 Standard deviation regularization 5](#_Toc527535302)

[1.4.4 Auto training variants quality cutoff 6](#_Toc527535303)

[1.5 Name Your Session 6](#_Toc527535304)

[1.6 Email Address 6](#_Toc527535305)

[2. View Landscapes 6](#_Toc527535306)

[2.1 Previously imputed maps 6](#_Toc527535307)

[2.2 View maps by session ID 6](#_Toc527535308)

[2.3 View maps by Uniprot ID 6](#_Toc527535309)

[3. Downloads 7](#_Toc527535310)

[3.1 Download Weile et al. 2017 7](#_Toc527535311)

[3.2 Download Data Template 7](#_Toc527535312)

[3.3 Download Help Document 7](#_Toc527535313)

[3.4 Download Supported Uniprot IDs 7](#_Toc527535314)

[4. Map Viewer 7](#_Toc527535315)

[4.1 Download CSV 7](#_Toc527535316)

[4.2 Download Figures 7](#_Toc527535317)

[4.3 PubMed Link 8](#_Toc527535318)

[4.4 View options / Legends 8](#_Toc527535319)

[4.4.1 Original Fitness 8](#_Toc527535320)

[4.4.2 Refined Fitness 8](#_Toc527535321)

[4.4.3 Polyphen Score 8](#_Toc527535322)

[4.4.4 SIFT Score 8](#_Toc527535323)

[4.4.5 Provean Score 9](#_Toc527535324)

[4.4.6 Allele Frequency 9](#_Toc527535325)

[4.5 Map Visualization 9](#_Toc527535326)

[4.5.1 Accessible Surface Area (ASA) track 9](#_Toc527535327)

[4.5.2 Secondary structure (SS) track 9](#_Toc527535328)

[4.5.3 Pfam domain track 9](#_Toc527535329)

[4.5.4 Variant tracks 10](#_Toc527535330)

[4.5.5 Pop Up Panel 10](#_Toc527535331)

[5. Appendix 10](#_Toc527535332)

[5.1 Raw data format 10](#_Toc527535333)

[5.2 Processed data format 11](#_Toc527535334)

[5.3 Imputation result data format 11](#_Toc527535335)

[5.4 References 13](#_Toc527535336)

Welcome to the Human Protein Variants Effect Map Imputation Toolkit User Guide. The purpose of this toolkit is to impute, refine and visualize data from multiplexed variant effect assays (MAVEs) to produce missense variant effect (VE) maps. Specifically, we provide: 1) a front-end web application (<http://impute.varianteffect.org/>, source: <https://github.com/joewuca/imputation>) that allows users to upload their own MAVE data and visualize or download a complete map of variant effects; and 2) a back-end data processing service that performs imputation and refinement. This user guide will focus on the front-end web application and proper and effective use of the toolkit. For users interested in the back-end data processing service, please see previous methods in Weile et al, (Molecular Systems Biology 2017) and other methodological references to be added here as the toolkit evolves to implement other methods.

The user guide is organized into four sections based on the user interface (see the purple boxes on the figure below): **(1) Upload and Impute:** for uploading input MAVE data, metadata and parameters, imputing missing values, and refining measured values. **(2) View Landscapes:** for selecting and visualizing the finished variant effect maps (either from the user or selected from previously-finished examples). **(3) Downloads:** for downloading related information such as data template and help files **(4) Map viewer:** for visualizing and downloading finished variant effect maps.

NOTE: Human protein variant effect maps (imputed or otherwise) are research tools and should be appropriately validated before clinical use.


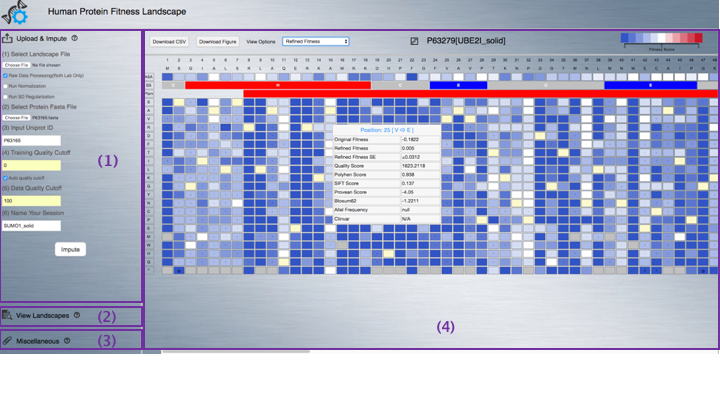


# Upload and impute

## Select Landscape File

### Raw Data (Checkbox)

There are two supported data formats for the input protein MAVE data:

- **Raw data format**

A raw landscape data format for human protein variant effect map calculation and imputation, developed for raw MAVE data from the TileSeq method (Welie *et.,al* 2017). Please refer to Section 5.1 for the details.

- **Processed data format**

The processed data format is a more general data format can be used for MAVE data generated from a variety of experimental approaches. Please refer to Section 5.2 for the details.

### Example – UBE2I and BRCA1


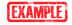


Clicking the example button will initiate download of a zip file with files needed for imputing the variant effect map of two example proteins (UBE2I; the human SUMO conjugase enzyme that recognizes sumoylation sites on client proteins and attaches the SUMO protein to these clients, data collected from Welie *et.,al* 2017. BRCA1; the human breast cancer type 1 susceptibility protein, data collected from Findlay *et.,al* 2018) will be downloaded to your local disk, and the default parameters for UBE2I imputation will be loaded as well. There are six files in the zip file:

- rawData_UBE2I.txt (UBE2I raw data)
- processedData_UBE2I.txt (UBE2I processed data)
- P63279_UBE2I.fasta (UBE2I full protein sequence)
- processedData_BRCA1-RING.txt (BRCA1 RING domain processed data)
- processedData_BRCA1-BRCT.txt (BRCA1 BRCT domain processed data)
- P38398_BRCA1.fasta (BRCA1 full protein sequence)

Before you hit the “impute” button to run the example protein imputation, please make sure you have loaded the raw/processed data and the fasta file, and also have correctly checked the raw data checkbox if you are using the raw data file as input (or left it unchecked if you are using the processed data input file). The running time for the example imputation is expected to be less than one minute. Please note clicking the example button will set the session name and Uniprot ID for UBE2I imputation, to impute BRCA1 you need to change the Uniprot ID to P38398 and also change your session name after loading the BRCA1 related input files.

## Select Protein Fasta File

Please select the protein fasta file that is consistent with your input data. For example, if your MAVE experiment was done on the non-canonical protein variant, your input fasta file should contain the protein sequence from this non-canonical variant.

## Input Uniprot ID

Please input the ID from UniProt (Bateman, A. *et al.,* 2017) that corresponds to your target protein to retrieve the precomputed protein features such as structure features, Polyphen2 (Adzhubei *et al.,* 2016), SIFT (Ng, P. C. & Henikoff, S 2011) and PROVEAN (Choi *et al.,* 2016) scores. We have precomputed features for the Uniprot human IDs that have at least one missense variant in ClinVAR (Landrum, M. J. *et al.,* 2018) database (Currently ~3200 proteins). The full list of supported Uniprot IDs can be downloaded, please check Section 3.4 for details. If you submit a valid human Uniprot ID that is not in our list, you will be given an option to request retrieval of protein features and notified by email when completed.

## Set Parameters

### Filter low quality missense variants

This option filters out input data for those missense variant records with a quality score below the defined quality score cutoff:

- **variant quality score cutoff**

The cutoff used for removing input data for variants for which MAVE data does not surpass a specified quality score.

### Data rescaling

The data rescaling option rescales the fitness of each variant such that the median of stop codon variants has fitness 0 after rescaling and the median of synonymous variants has fitness 1 after rescaling. We set the variant fitness score to 0 If the score is smaller than 0 after rescaling. If your processed data is already rescaled to your preference, you should uncheck the “Data rescaling” checkbox. If the box is checked, you can set up the following two parameters:

- **Synonymous/nonsense variant quality cutoff**

This cutoff is used for filtering out the low quality synonymous and nonsense variants that you do not want to include for median calculation. The default value is 0.

- **Nonsense variant exclusion regions**

Although it is often assumed that nonsense variants are severe null mutations, this is not necessarily the case, especially for nonsense variants near the 3’ end of the original open reading frame, i.e., near the wild-type stop codon. You can define the regions of the protein for which nonsense variants should be excluded in the median calculation used for rescaling. The input format is “a:b” to exclude a single region from position a to position b (e.g. to exclude nonsense variants in the last 10% of the wild type open reading frame for a 100 amino acid protein, use “90:100”. In rare cases it may be desired to exclude multiple regions. This can be done by concatenating inputs (e.g. “a:b,c:d”). The default value is “0:0”.

### Standard deviation regularization

This option regularizes the measured standard deviation (SD) with a prior estimate of SD calculated from an overall regression of SD values against fitness values when the number of replicates is small. This Bayesian regularization procedure is based on Baldi, P. & Long *et al.,* 2001.

- **Number of replicates required to skip standard deviation regularization**

When an insufficient number of replicates is available to accurately measure SD, it is appropriate to use regularization to smooth observed data and ‘nudge’ SD estimates towards prior expectation for the SD. This parameter defines a minimum number of replicates, *k*. If the user provides *j* observed replicates and this is lower than *k*, a regularized estimate of *σ* is calculated as in Weile *et al.,* 2017, effectively giving weight *j* / *k* to the observed data. Larger values of *k* give more weight on the prior SD estimate during regularization. The default value for *k* is 8.

### Auto training variants quality cutoff

The auto training quality cutoff is determined by searching for the training data cutoff that gives the best performance in terms of predicting the test dataset (which consists of the top 20% of variants, ranked by quality score). If this option is unchecked, then the cutoff must be set manually:

- **Training variants quality cutoff**

Manually set the quality score cutoff to remove low-quality variants from the imputation training variant examples.

## Name Your Session

You can name your current imputation session with any string such as the protein name, datetime etc. The final session ID is set as “[Uniprot ID][Name Your Session string]”. Once the imputation is done, you can visualize the result on the map viewer and also revisit your results through View Landscapes using your session ID.

## Email Address

You can input your email address to receive an email alert when the imputation job is done. The email will provide your session ID and links to download results.

# View Landscapes

## Previously imputed maps

Select a map from previously imputed variant effect maps provided using the list box.

## View maps by session ID

To view your previously-imputed variant effect maps, input your session ID and click View.

## View maps by Uniprot ID

To view maps for other predictors (Polyphen2, SIFT and Provean), input the Uniport ID of interest and click View. Note this function is only available for the Uniprot IDs supported by our system (See section 3.4). The variant effect maps for the input Uniprot ID will show if they have been previously imputed in our system.

# Downloads

## Download Weile et al. 2017

The button links to the script, the original and imputed maps from Weile et al., 2017.

## Download Data Template

The input data templates (raw and processed). Please do not change the column names as these are used for parsing the input file.

## Download Help Document

This button downloads this document in pdf format.

## Download Supported Uniprot IDs

This button downloads a full list of supported Uniprot IDs so far. The Boolean value for each column indicates the availability of the corresponding feature.

# Map Viewer

## Download CSV

Download the imputed result in CSV format (see Section 5.3 for a description of each column).

## Download Figures

Two figures can be downloaded: one figure shows the original input variant effect map with missing values and the other shows the finished (imputed and refined) variant effect map. Each is provided in PDF format. When the Download Figures button is clicked, two separate web pages should open, one for each figure. Some browsers may have settings such that the second webpage is blocked. In this case, please alter browser settings to enable popup windows for this web application. The figures are similar but not identical to the html-encoded images provided in the visualization window.

## PubMed Link

Link to the paper in which the previously imputed variant effect map was published.

## View options / Legends

### Original Fitness

Original Fitness option visualizes your input variants (after rescaling. The original fitness score range is 0 to 2 with 1 indicating wild-type (WT) fitness.


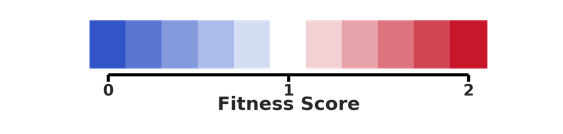


### Refined Fitness

Refined Fitness option visualizes the variants effect map after imputation and refinement. The refined fitness core range is 0 to 2 with 1 indicating WT fitness.


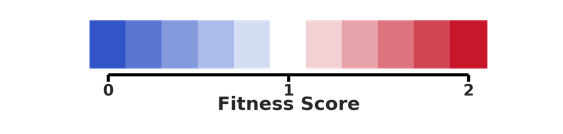


### Polyphen Score

Polyphen Score option visualizes the variants effect map using polyphen2 score (http://genetics.bwh.harvard.edu/pph2/). The polyphen2 score range is 0 to 1 representing the estimated probability that the variant has a damaging impact on protein function.


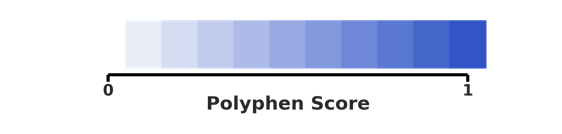


### SIFT Score

SIFT Score option visualizes the variants effect map using SIFT score (http://sift.jcvi.org/). The SIFT score range is 0 to 1 representing the probability of being tolerated. Variants with normalized probabilities less than 0.05 are predicted to be deleterious, those greater than or equal to 0.05 are predicted to be tolerated.


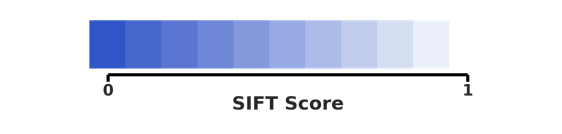


### Provean Score

Provean Score option visualises the variants effect map using Provean score (<http://provean.jcvi.org/>). The Provean score range is -13 to 4. If the Provean score is equal to or below a predefined threshold (-2.5), the protein variant is predicted to have a "deleterious" effect. If the Provean score is above the threshold, the variant is predicted to have a "neutral" effect.


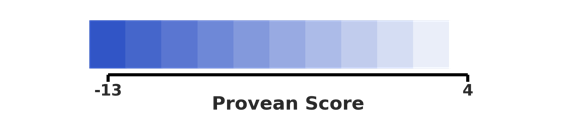


### Allele Frequency

The Allele Frequency option visualizes the variants effect map using the -log10(MAF) score, where MAF is minor allele frequency as scored in the GNOMAD database (http://gnomad.broadinstitute.org/). The -log10 MAF score ranging from 0.3 to 10.


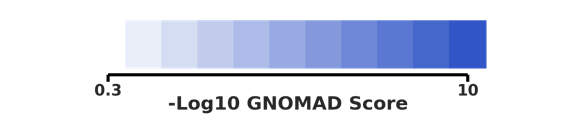


## Map Visualization

### Accessible Surface Area (ASA) track

The ASA track shows the relative Accessible Surface Area of each position of the protein, which is retrieved from a PDB structure of the input protein, where available. The ASA score ranges from 0 to 1, where 1 indicates the highest accessible surface area value.


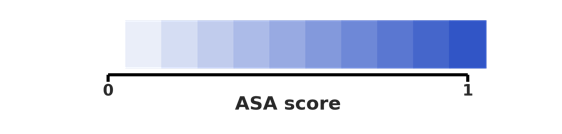


### Secondary structure (SS) track

The SS track shows the secondary structure for each position of the protein. The SS data is retrieved using PsiPred (<http://bioinf.cs.ucl.ac.uk/psipred/>). More specifically, the letters C, H, and E indicate coiled coil, alpha helix and beta sheet respectively.

### Pfam domain track

The Pfam track shows the Pfam domain of the protein. The Pfam domain boundaries are retrieved from Pfam database (<https://pfam.xfam.org/>). Clicking the domain name in the track leads to the Pfam webpage of that domain.

### Variant tracks

There are 21 variant tracks, representing the 20 possible amino acid values plus the stop codon. The color code and value for each entry of variant tracks depend on the current selected view options. For wild type entries, where the amino acid track corresponds to the wild type amino acid, the color code is YELLOW (this is not shown on the fitness score legend but is by definition fitness 1). The small vertical line in each entry represents the standard error of the fitness score.

### Pop Up Panel

When you mouse over each entry, you will see a popup panel showing the detailed information of that entry for all view options.

# Appendix

## Raw data format

| Column Name | Description |
| --- | --- |
| wt_aa | The wild type residue of the variant |
| Pos | The position of the variant |
| mut_aa | The substituted residue of the variant |
| wt_codon | The wild type codon of the variant |
| mut_codon | The substituted codon of the variant |
| Annotation | NONSYN/SYN/STOP |
| nonselect | The variant allele frequency in variant pool under non-selective condition |
| select | The variant allele frequency in variant pool under selective condition |
| controlNS | The apparent variant allele frequency in control(wt) template under non-selective condition (a control in which all variants are sequencing errors) |
| controlS | The apparent variant allele frequency in control(wt) template under selective condition (a control in which all variants are sequencing errors) |
| replicate_id | The id for replicates. Please make sure different replicate has different replicate id. |

## Processed data format

| Column Name | Description |
| --- | --- |
| aa_ref | The wild type residue of the variant |
| aa_pos | The position of the variant |
| aa_alt | The substituted residue of the variant |
| quality_score | The quality score of the variant. For example, the allele frequency of the variant in non-selective condition. |
| num_replicates | The number of replicates used to calculate the fitness score. |
| fitness_input | The original fitness score of a variant (mean taken over all replicates) |
| fitness_input_sd | The standard deviation of original fitness scores across all replicates. |

## Imputation result data format

| Column Name | Description |
| --- | --- |
| aa_ref | The wild type residue of the variant |
| aa_pos | The position of the variant |
| aa_alt | The substituted residue of the variant |
| annotation | STOP/SYN/NONSYN |
| quality_score | The quality score of the variant |
| num_replicates | Number of replicates |
| pseudo_counts | The pseudocount of the replicates used for standard deviation Bayesian regularization |
| fitness_input | The original fitness score of a variant (mean taken over all replicates) |
| fitness_input_sd | The standard deviation of original fitness scores across all replicates. |
| fitness_org | The rescaled fitness of the variant |
| fitness_sd_org | The standard deviation of the rescaled variant fitness values |
| fitness | The reversed and floored fitness_org |
| fitness_sd | The rescaled fitness standard deviation of the variant (same as fitness_org_sd) |
| fitness_sd_prior | The prior (background) fitness standard deviation of the variant, derived from regression (fitness_sd against fitness) |
| fitness_sd_reg | The regularized standard deviation of variant fitness |
| fitness_se_reg | The regularized standard error of variant fitness |
| fitness_imputed | The imputed fitness of the variant |
| fitness_imputed_se | The standard error of the imputed variant fitness value (\|fitness_imputed – fitness\|) |
| fitness_imputed_se_prior | The prior estimate of standard error used in imputing the standard error of variant fitness, derived from regression (fitness_imputed_se against fitness_imputed) |
| fitness_refine | The refined fitness of the variant, achieved by combining fitness and fitness_imputed (a weighted average with weighting by inverse of estimated variance) |
| fitness_refine_se | The standard error estimate for the refined variant fitness value |
| Polyphen_score | The polyphen2 score of the variant |
| Sift_score | The SIFT score of the variant |
| Provean_score | The Provean score of the variant |
| gnomad_af | The Gnomad-derived minor allele frequency of the variant |
| asa_mean | The accessible surface area of the variant position |
| aa_psipred | The secondary structure of the SS segment starting from aa_position to ss_end_pos (C: Coiled coil, H: Alpha Helix, E: Beta Sheet). The value only exists on the STOP variants record to avoid redundancy as there are 22 records for each position (1 STOP, 1 SYN, 20 NONSYN) |
| ss_end_pos | The end position of the SS segment starting from aa_position |
| hmm_id | The HMM id of the Pfam domain starting from aa_position to pfam_end_pos. The value only exists on the STOP variants record to avoid redundancy as there are 22 records for each position (1 STOP, 1 SYN, 20 NONSYN) |
| pfam_end_pos | The end position of Pfam domain starting from aa_position |

## References

Adzhubei, I et al., Predicting functional effect of human missense mutations using PolyPhen-2. Curr. Protoc. Hum. Genet. Chapter 7, Unit7.20 (2013).

Baldi, P., & Long, A Bayesian framework for the analysis of microarray expression data: regularized t -test and statistical inferences of gene changes. Bioinformatics, 17(6), 509–519 (2001).

Bateman, A. et al. UniProt: The universal protein knowledgebase. Nucleic Acids Research45, D158–D169 (2017)

Choi, Y et al., Predicting the functional effect of amino acid substitutions and indels. PLoS One 7, e46688 (2012).

Findlay, G. M. et al. Accurate classification of BRCA1 variants with saturation genome editing. Nature (2018). doi:10.1038/s41586-018-0461-z

Henikoff, S. & Henikoff, J. G. Amino acid substitution matrices from protein blocks. Proceedings of the National Academy of Sciences 89, 10915–10919 (1992).

Landrum, M. J. et al. ClinVar: Improving access to variant interpretations and supporting evidence. Nucleic Acids Research 46, D1062–D1067 (2018).

Ng, P. C. & Henikoff, S. Predicting deleterious amino acid substitutions. Genome Research11, 863–874 (2001).

Weile et al., A framework for exhaustively mapping functional missense variants. Mol Syst Biol 13: 957 (2017)
