# Supplementary material for: A web application and service for imputing and visualizing missense variant effect maps
Source: Bioinformatics. 2019 Jan 14;35(17):3191–3. doi: 10.1093/bioinformatics/btz012 (PMC6735881; doi:10.1093/bioinformatics/btz012)
Supplement: btz012_Supplementary_Data [file btz012_supplementary_data.zip › btz012-suppl_data/imputation_installation_guide_2018_12_04.docx]

**Imputation web application setup guide**

Welcome to the imputation web application setup guide. The purpose of this guide is to demonstrate how to build the imputation web application from scratch. The imputation web application is embedded in a customized Docker image (https://www.docker.com/), which can be hosted on any publicly accessible server. In our case it is a VM instance on the Google Cloud platform. Details on how to host Docker image on a Google VM instance can be found via this link <https://cloud.google.com/compute/docs/containers/deploying-containers>. This guide assumes you already have a publicly accessible server.

There are three main steps to set up the imputation web application:

1. **Build Docker image with Apache web server and Python support**
2. **Deploy the front-end and back-end scripts**
3. **Run Docker image on the publicly accessible server**

Here are the details for each step:

1. **Build Docker image with Apache web server and Python support.**

Please follow the instructions on <https://docs.docker.com/> to install Docker on your own system, then copy <https://github.com/joewuca/imputation/tree/master/imputation/docker> folder to your system. Under the Docker folder, please run command “*docker build -t YOUR_DOCKERIMAGENAME”* to create the Docker image configured by the Dockerfile under the same folder. The newly-built Docker image comes equipped with Apache web server (with WSGI support) and Python modules needed for the imputation pipeline to run properly.

1. **Deploy the front-end and back-end script**

The front-end application was developed using Google Web Toolkit (<http://www.gwtproject.org/>) which allows user to write web applications in Java and compile them to JavaScript. The back-end script is written in Python. To deploy the front-end and back-end scripts, first create two folders (“projects” and “database”) on your publicly accessible server. Copy everything in https://github.com/joewuca/imputation to your “projects” folder. Download http://impute.varianteffect.org/downloads/database.tar.gz and unzip it to your “database” folder. Here is a brief description of the purpose of each sub folder:

- **projects/imputation/gwt/src:** front-end Java source code (Google Web Toolkit).
- **projects/imputation/gwt/www:** front-end complied Javascript and resource files.
- **projects/imputation/python:** back-end Python source code for imputation.
- **projects/ml/python:** back-end Python source code for machine learning problem.
- **database/humandb/dms/features:** pre-computed imputation features for supported proteins such as secondary structure, SIFT, PolyPhen2 and Provean.
- **database/humandb/dms/other_features:** other general features such as amino acid chemical physical properties.

1. **Run Docker image on the publicly accessible server**

After front-end and back-end script deployment, please run the following Docker command to launch the imputation web application:

*“docker run -dit --name imputation -p 80:80 -v PATH_TO_projects_FOLDER:/usr/local/projects -v PATH_TO_database_FOLDER:/usr/local/database YOUR_DOCKERIMAGENAME”.*

This command maps the “projects” and “database” folder on the publicly accessible server to the corresponding folders in your Docker image, and also passes all the incoming http requests on port 80 to the Docker image.
