# Supplementary material for: A web application and service for imputing and visualizing missense variant effect maps
Source: Bioinformatics. 2019 Jan 14;35(17):3191–3. doi: 10.1093/bioinformatics/btz012 (PMC6735881; doi:10.1093/bioinformatics/btz012)
Supplement: btz012_Supplementary_Data [file btz012_supplementary_data.zip › btz012-suppl_data/imputation_supplementary_2018_12_04.docx]

Supplementary information

**Supplementary Figure 1:** The coverage of the five MAVE experiments from [Weile *et al.,* 2017]. The coverage is defined as the fraction of all possible amino acid changes (19 possible missense variants and the stop codon) that were considered measured in the MAVE map prior to imputation.

**Supplementary Figure 2:** The imputation performance on protein UBE2I using five different machine learning methods. The performance is defined as 10 cross validation RMSE. Graident Boost Tree is the best and also is the best on other four proteins (SUMO1, TPK1, CALM1 and CBS)

**Supplementary Figure 3:** Feature importance measures (derived from imputation of UBE2I using Gradient Boosted Trees). Only the first ten most important features are included. Feature importance in the imputation model for the other four proteins (SUMO1, TPK1, CALM1 and CBS) yielded ranked feature importance similarly, with average fitness at each position being the most important feature in all cases. The description of each feature can be found in Supplement Table 1.

**Supplementary Figure 4:** Evaluation of model error for the UBE2I variant effect map imputation using different training data quality score thresholds. The quality socre here is defined as the initial variant abundence in non-selective condition in our experiment. The y-axis shows RMSE values in terms of predicting the test dataset which consists of the top 20% of variants ranked by quality score. Here the best quality score cutoff was ~100.

Supplementary Table 1: Features used for training imputation models

| Feature Category | Feature Name | Description |
| --- | --- | --- |
| Engineered feature | average_pos_fitness_score | Average fitness value at each position |
| Scores from other computational predictors | blosum100 | BLOSUM100 (Henikoff, S. & Henikoff, J. G. 1992) |
|  | polyphen_score | PolyPhen-2 (Adzhubei *et al*., 2016) |
|  | provean_score | PROVEAN (Choi *et al*., 2016) |
|  | sift_score | SIFT (Ng, P. C. & Henikoff, S 2011) |
| Chemical and physical properties  (wild type and substituted residues) | mw_ref, mw_alt | Molecular weight |
|  | pka_ref, pka_alt | Acid dissociation constant $\boldsymbol{p}\boldsymbol{K}_{\boldsymbol{a}}$ |
|  | pkb_ref, pkb_alt | Base dissociation constant $\boldsymbol{p}\boldsymbol{K}_{\boldsymbol{b}}$ |
|  | pi_ref, pi_alt | Isoelectronic point $\boldsymbol{pI}$ |
|  | hi_ref, hi_alt | Hydropathy index |
|  | vadw_ref, vadw_alt | Van der Waals volume |
|  | cyclic_ref, cyclic_alt | Cyclic |
|  | positive_ref, positive_alt | Positive Charge |
|  | negative_ref, negative_alt | Negative Charge |
|  | hydrophobic_ref, hydrophobic_alt | Hydrophobic |
|  | polar_ref, polar_alt | Polar |
|  | ionizable_ref, ionizable_alt | Ionizable |
|  | aromatic_ref, aromatic_alt | Aromatic |
|  | aliphatic_ref, aliphatic_alt | Aliphatic |
|  | hbond_ref, hbond_alt | Hydrogen bond |
|  | sulfur_ref, sulfur_ref | Sulfur containing |
|  | essential_ref, essential_alt | Essentiality to human |
| Structural and Domain properties | aa_psipred_encode | Secondary structure |
|  | in_domain | In/out pFam domain |
|  | asa_mean | Solvent accessible surface area |
| Other features | aa_pos | Position of the residue |

Supplementary Reference

Adzhubei, I et al., Predicting functional effect of human missense mutations using PolyPhen-2. Curr. Protoc. Hum. Genet. Chapter 7, Unit7.20 (2013).

Choi, Y et al., Predicting the functional effect of amino acid substitutions and indels. PLoS One 7, e46688 (2012).

Henikoff, S. & Henikoff, J. G. Amino acid substitution matrices from protein blocks. Proceedings of the National Academy of Sciences 89, 10915–10919 (1992).

Ng, P. C. & Henikoff, S. Predicting deleterious amino acid substitutions. Genome Research11, 863–874 (2001).

Weile et al., A framework for exhaustively mapping functional missense variants. Mol Syst Biol 13: 957 (2017)
